# Genome-Wide Local Ancestry Approach Identifies Genes and Variants Associated with Chemotherapeutic Susceptibility in African Americans

**DOI:** 10.1371/journal.pone.0021920

**Published:** 2011-07-06

**Authors:** Heather E. Wheeler, Lidija K. Gorsic, Marleen Welsh, Amy L. Stark, Eric R. Gamazon, Nancy J. Cox, M. Eileen Dolan

**Affiliations:** 1 Section of Hematology/Oncology, Department of Medicine, University of Chicago, Chicago, Illinois, United States of America; 2 Department of Human Genetics, University of Chicago, Chicago, Illinois, United States of America; 3 Section of Genetic Medicine, Department of Medicine, University of Chicago, Chicago, Illinois, United States of America; Institut Jacques Monod, France

## Abstract

Chemotherapeutic agents are used in the treatment of many cancers, yet variable resistance and toxicities among individuals limit successful outcomes. Several studies have indicated outcome differences associated with ancestry among patients with various cancer types. Using both traditional SNP-based and newly developed gene-based genome-wide approaches, we investigated the genetics of chemotherapeutic susceptibility in lymphoblastoid cell lines derived from 83 African Americans, a population for which there is a disparity in the number of genome-wide studies performed. To account for population structure in this admixed population, we incorporated local ancestry information into our association model. We tested over 2 million SNPs and identified 325, 176, 240, and 190 SNPs that were suggestively associated with cytarabine-, 5′-deoxyfluorouridine (5′-DFUR)-, carboplatin-, and cisplatin-induced cytotoxicity, respectively (p≤10^−4^). Importantly, some of these variants are found only in populations of African descent. We also show that cisplatin-susceptibility SNPs are enriched for carboplatin-susceptibility SNPs. Using a gene-based genome-wide association approach, we identified 26, 11, 20, and 41 suggestive candidate genes for association with cytarabine-, 5′-DFUR-, carboplatin-, and cisplatin-induced cytotoxicity, respectively (p≤10^−3^). Fourteen of these genes showed evidence of association with their respective chemotherapeutic phenotypes in the Yoruba from Ibadan, Nigeria (p<0.05), including *TP53I11*, *COPS5* and *GAS8*, which are known to be involved in tumorigenesis. Although our results require further study, we have identified variants and genes associated with chemotherapeutic susceptibility in African Americans by using an approach that incorporates local ancestry information.

## Introduction

Genome-wide association (GWA) studies have been successful in identifying common genetic variants associated with many diseases and traits [1]. Nearly 90% of these studies have been completed in populations of European ancestry [2]. Technical reasons for the paucity of GWA studies in African populations include increased population structure, which reduces effective sample size, and reduced linkage disequilibrium (LD), which means genotyped SNPs do not tag as many loci as in populations with larger LD-blocks [3]. Studies in populations of recent African ancestry are crucial because additional variants present at higher frequencies in African populations may be absent or rare in European populations. It is unclear whether associations found in European populations can be consistently replicated in African populations: decreased linkage disequilibrium (LD) and gene by environment interactions could contribute to lack of replication [4]. Importantly, the inclusion of populations of diverse ancestry in genomic studies advances the goal of reducing health disparities [3]. We chose to address these issues by investigating the genetics of chemotherapeutic susceptibility in an African American population using both SNP- and gene-based genome-wide approaches.

Previous studies have shown that individual response to chemotherapy is partially due to genetic factors with heritability estimates ranging from 0.3–0.4 [5]. Performing GWA studies for chemotherapeutic response in a clinical setting is challenging due to a number of confounders such as diet and concomitant medications. Even more challenging is to obtain a large clinical study population of African Americans treated with the same dosage regimen that have been systematically evaluated for response and toxicity. To overcome these challenges, we have developed a cell-based model that employs Epstein Barr virus (EBV)-transformed lymphoblastoid cell lines (LCLs) from different world populations as useful discovery tools in genetic studies of chemotherapeutic susceptibility [6]–[10]. Others have claimed non-genetic factors such as baseline growth rate, EBV copy numbers and ATP levels may influence drug-induced phenotypes in LCLs [11]. We have shown that growth rate does associate with chemotherapeutic-induced cytotoxicity and should be considered in all LCL analyses, but baseline EBV copy numbers and ATP levels do not associate with chemotherapeutic-induced cytotoxicity [12]. Some SNPs associated with chemotherapeutic susceptibility in LCL discovery studies have recently been successfully replicated in patient populations by associating with phenotypes like tumor response and overall survival, which demonstrates the utility of this model [13], [14]. In this study, we expand our model to cell lines derived from African Americans.

We chose to focus on four distinct chemotherapeutic drugs, cytarabine, capecitabine, carboplatin and cisplatin, because of their use and importance in the treatment of African Americans. Cytarabine is an antimetabolite and the mainstay of treatment for acute myeloid leukemia (AML) [6]. In some AML patients, resistance to cytarabine is a major reason for treatment failure and in others, cytarabine is associated with several adverse side effects, including myelosuppression and neurotoxicity [15]–[19]. In a study by the Children's Oncology Group of pediatric patients with AML, patients with African ancestry had significantly worse survival compared with patients of exclusively European ancestry [20]. Capecitabine, an antimetabolite pro-drug commonly used to treat breast and colon cancers, is associated with two well-described toxicities: diarrhea and hand-foot syndrome, which often result in treatment delays or dose-reductions [21], [22]. Capecitabine is often used to treat triple negative breast cancer, which disproportionately affects individuals of African ancestry [23]. Capecitabine is also used to treat advanced stage colon cancer and outcomes are worse for African Americans than Caucasians [24]. Carboplatin and cisplatin are platinating chemotherapy agents commonly used to treat lung, testicular, head and neck, colorectal and gynecological cancers [25]–[29]. Both agents are associated with particular toxicities, predominantly myelosuppression for carboplatin and nephrotoxicity and ototoxicity for cisplatin [30]–[32]. There is a higher mortality rate reported for lung and gynecological cancers in African Americans compared with Caucasians [33], both of which are commonly treated with platinating agents [7]. Many of these outcome disparities are likely influenced by socioeconomic factors, but genetic variation is also thought to contribute [2], [3].

LCLs from the International HapMap Project's [34] African American population from the Southwestern United States (ASW) were used to perform genome-wide SNP- and gene-based association studies for susceptibility to cytarabine, carboplatin, cisplatin and 5′-DFUR (LCLs do not express cytidine deaminase, therefore we used 5′-DFUR, which is produced from capecitabine by the action of this enzyme). To account for population structure in this admixed population, we incorporated local ancestry information into our association model. Our local ancestry approach differs from the traditional global ancestry approach because it considers ancestry at each particular chromosomal locus. Thus, in addition to identifying potential variants and genes involved in chemotherapeutic drug response in African Americans, our study contributes to understanding the complex trait genetics of other phenotypes in any admixed population. We identified SNPs associated with each of the four drug phenotypes and an enrichment of carboplatin-associated SNPs in the top cisplatin-associated SNPs. Using a recently developed gene-based GWA approach [35], we identified 98 suggestive candidate genes for chemotherapeutic susceptibility in the ASW LCLs (p≤10^−3^). This set included 14 genes that showed evidence of replication in the Yoruba population from Ibadan, Nigeria (YRI, p<0.05). We also show that many of the associated SNPs identified in the ASW are not polymorphic in populations of European ancestry, which highlights the need for genetic studies in diverse populations.

## Results

### Cytotoxicity phenotypes

Lymphoblastoid cell lines (LCLs) from 83 ASW individuals were evaluated for cellular susceptibility to four chemotherapeutic drugs (cytarabine, 5′-DFUR, carboplatin and cisplatin) using a short-term cell growth inhibition assay. For carboplatin and cisplatin, the percent survival data were used to calculate the concentration at which 50% growth inhibition occurred (IC_50_) for each cell line. The mean (±SD) IC_50_ was 20.1 (±7.1 µM) for carboplatin and 4.6 (±4.0 µM) for cisplatin. However, in evaluating cellular sensitivity to cytarabine and 5′-DFUR treatment, some resistant cell lines did not result in 50% cell growth inhibition, therefore the area under the survival-concentration curve (AUC) was calculated for each cell line. The mean (±SD) AUC was 1791 (±335 [%•µM]) for cytarabine and 2619 (±353 [%•µM]) for 5′-DFUR. For drugs with both measurements, AUC is highly correlated with IC_50_ (Spearman's rho = 0.98 for cisplatin and rho = 0.97 for carboplatin). Therefore, GWA results using different phenotypes (either IC_50_ or AUC) can be compared.

### Global ancestry vs. local ancestry in African Americans

**Figure 1:**
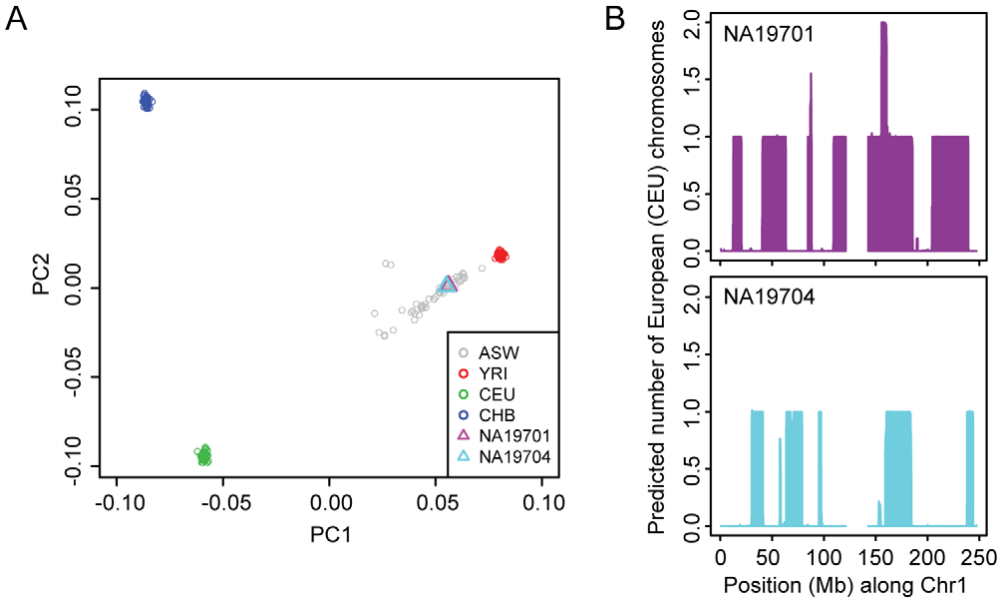
Global ancestry and local ancestry estimates. (A) The first two principal components (PCs), a measure of global ancestry, are plotted from the results of the genotype comparison of unrelated individuals in the four populations using EIGENSTRAT [37]. Two ASW individuals (NA19701 and NA19704) have similar PCs. (B) Shown are the predicted numbers of European chromosome copies, a measure of local ancestry, from the HAPMIX analysis [38] at each SNP locus for ASW individuals NA19701 and NA19704 at chromosome 1.

The ASW is a recently admixed population containing some chromosomal segments of African ancestry and some chromosomal segments of European ancestry. On average, African American genetic ancestry is 80% African and 20% European, but a wide range of percentages have been observed [36]. One approach to correct for population structure in admixed populations is to compute principal components (PCs) to model ancestry differences and use the significant PCs as covariates in the analysis. We used the EIGENSTRAT method to compute principal components by comparing ASW genotypes to HapMap reference populations YRI, CEU (Northern and Western European descent from Utah), and CHB (Han Chinese from Beijing) [37]. While the three reference populations cluster tightly when the first two principal components are plotted, the ASW individuals are more variable, mainly along the YRI-CEU axis (Figure 1A).

The EIGENSTRAT PC approach measures global ancestry across the entire genome and does not measure the ancestry at a particular chromosomal locus. To detect chromosomal segments of distinct ancestry in admixed populations, we employed HAPMIX, a new algorithm developed by Price et al. [38]. HAPMIX allowed us to estimate the number of African and European chromosomes in each ASW individual at each SNP locus, using phased YRI and CEU data as the reference parental populations. The number and size of European ancestry blocks varied among ASW individuals. Therefore, individuals with similar PCs, like NA19701 and NA19704 (Figure 1A), may have very different local ancestry patterns (Figure 1B). We defined the local ancestry as the predicted number of European ancestry chromosomes (0–2) at each SNP locus. To better correct for possible population stratification effects, the local ancestry at a particular SNP was used as a covariate in the association test for that SNP.

### SNP associations with chemotherapeutic-induced cytotoxicity

**Figure 2:**
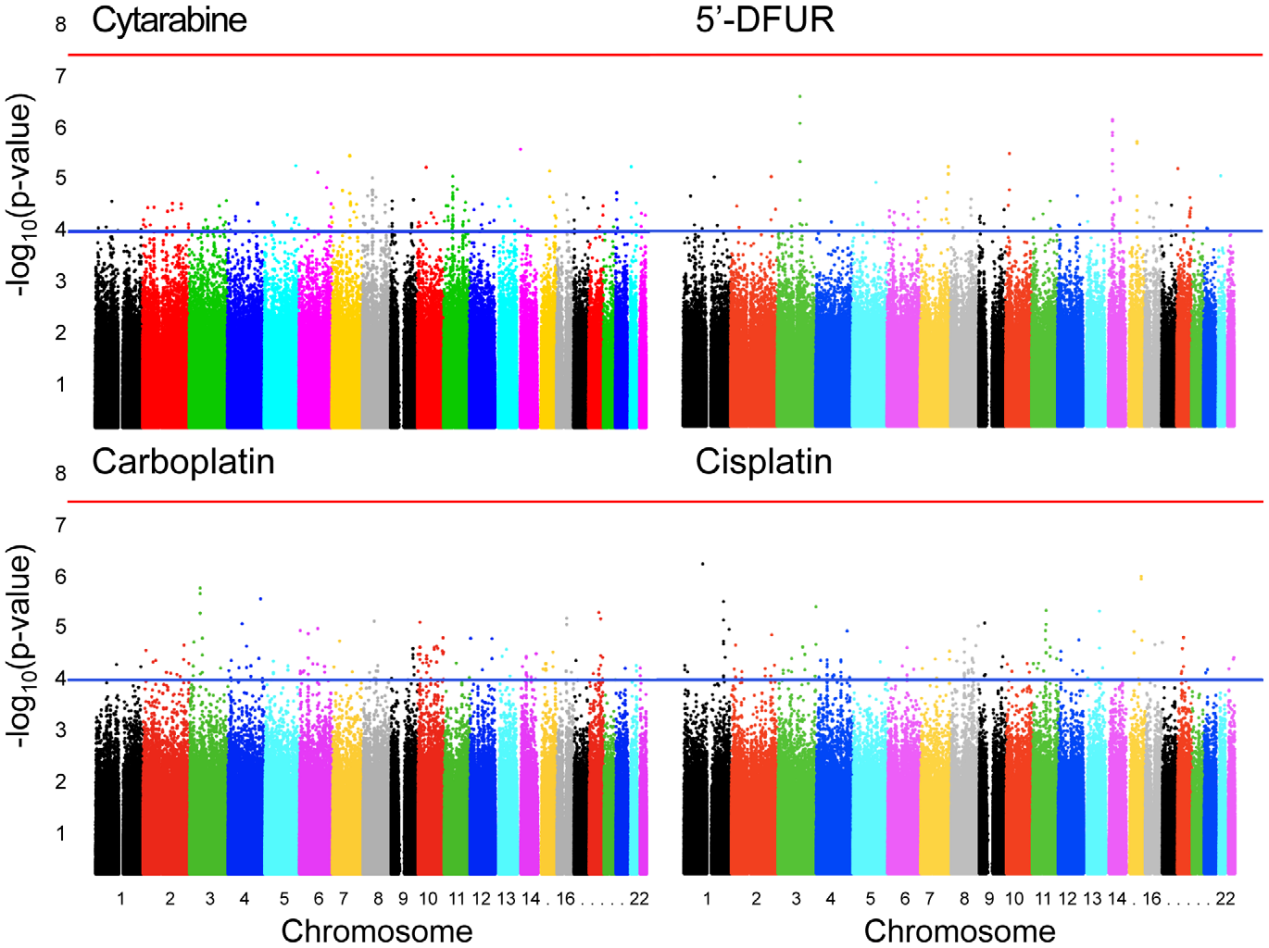
Genome-wide association results for chemotherapeutic-induced cytotoxicity in the ASW. Each point represents a SNP. Blue lines are at the suggestive significance threshold of p = 10^−4^. Red lines are at the genome-wide significance threshold of p = 5×10^−8^.

Using local ancestry covariates to account for possible population structure, we performed GWA studies between over 2 million SNPs in the ASW population and each drug-induced cytotoxicity phenotype. The phenotypes were adjusted for growth rate and the residuals were rank-transformed to normal prior to the association tests. At the suggestive significance threshold of p≤10^−4^, 325 SNPs were associated with cytarabine AUC, 176 with 5′-DFUR AUC, 240 with carboplatin IC_50_, and 190 with cisplatin IC_50_ (Figure 2). We chose p≤10^−4^ as suggestive because previous work has shown that SNPs associated with chemotherapeutic drug susceptibility at this p-value threshold and below are enriched for expression quantitative trait loci (eQTLs), an important functional class [39]. Table S1 lists all ASW drug-induced cytotoxicity-associated SNPs with p≤10^−4^.

**Figure 3:**
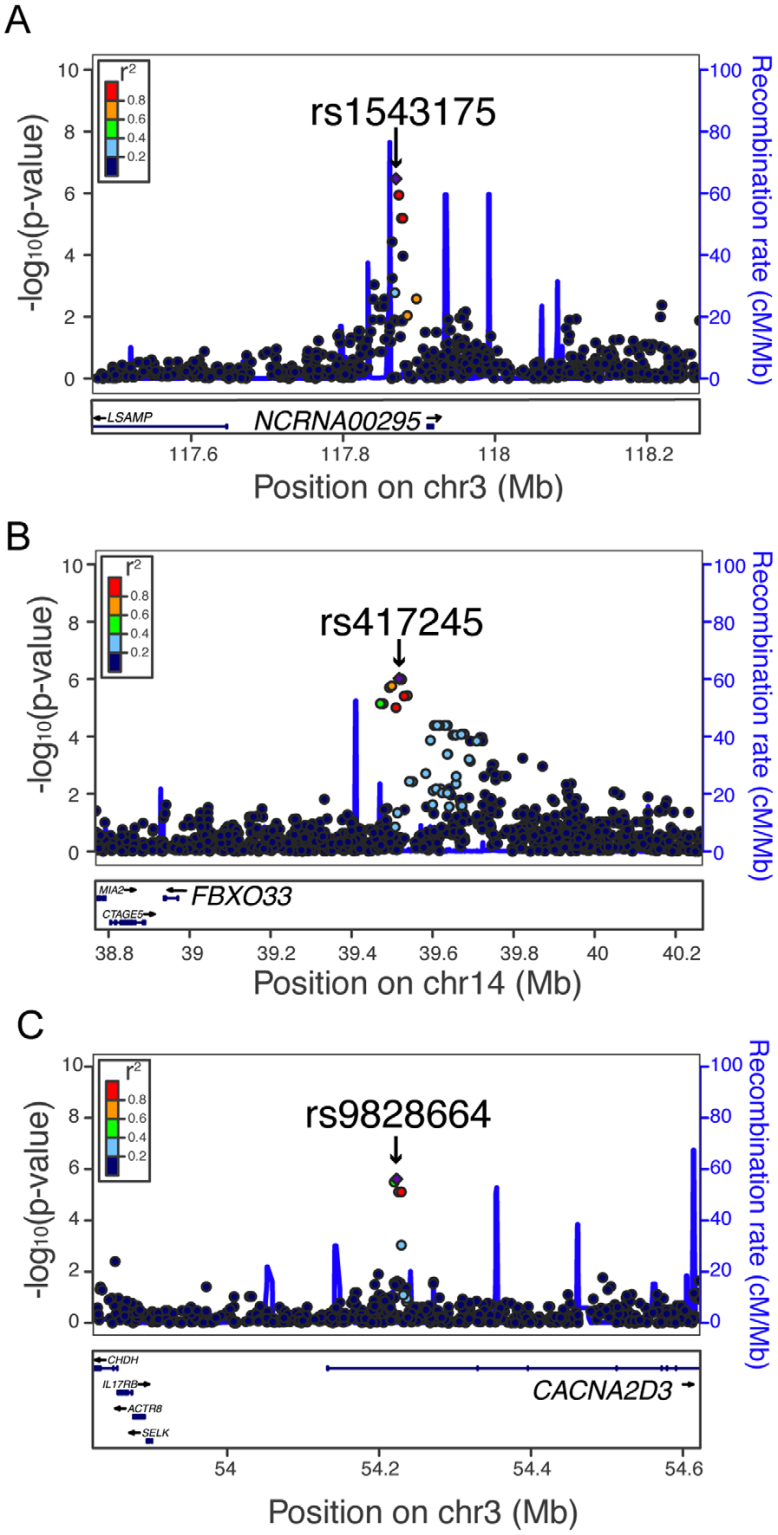
Genomic regions associated with chemotherapeutic-induced cytotoxicity in the ASW population. (A) rs1543175, which is 42 kb upstream of non-protein coding RNA 295 (*NCRNA00295*), associated with ASW 5′-DFUR AUC (p = 3.4×10^−7^) and is a *trans* eQTL in YRI (Table S1). (B) rs417245, which is 546 kb upstream of F-box only protein 33 (*FBXO33*), associated with ASW 5′-DFUR AUC (p = 9.6×10^−7^) and the expression of two genes in YRI (Table S1). (C) The SNP rs9828664 in the first intron of calcium channel, voltage-dependent, alpha 2/delta subunit 3 (*CACNA2D3*) associated with ASW carboplatin IC_50_ (p = 2.5×10^−6^) and the expression of two genes in YRI (Table S1). Plots were made with LocusZoom [67] and the color of each dot represents the SNP's linkage disequilibrium r^2^ in the YRI with the labeled SNP (purple diamond).

Although we have not generated gene expression data in the ASW, we determined if cytotoxicity-associated SNPs were eQTLs in the YRI, the most closely related population for which we have expression data (see *cis*- and *trans*-acting targets in Table S1). Several top SNPs for each drug were previously reported to be eQTLs in the YRI population [40]. Examples include rs1543175, which is 42 kb upstream of non-protein coding RNA 295 (*NCRNA00295*), is associated with ASW 5′-DFUR AUC (Figure 3A, p = 3.4×10^−7^), and is a *trans*-acting eQTL for IKAROS family zinc finger 1 (*IKZF1*) in YRI (Table S1, p = 2×10^−5^). An intergenic SNP, rs417245, is associated with ASW 5′-DFUR AUC (Figure 3B, p = 9.6×10^−7^) and is a *trans*-acting eQTL for 5-oxoprolinase (*OPLAH*) and 1-acylglycerol-3-phosphate O-acyltransferase 4 (*AGPAT4*) in YRI (Table S1, p≤10^−4^). An example within a gene is rs9828664, a SNP in the first intron of calcium channel, voltage-dependent, alpha 2/delta subunit 3 (*CACNA2D3*) that associated with ASW carboplatin IC_50_ (Figure 3C, p = 2.5×10^−6^) and the expression of two genes in YRI (Table S1). One rs9828664 gene expression association is *cis*-acting (filamin B, beta, *FLNB*, p = 8×10^−3^) and one is *trans*-acting (family with sequence similarity 91, member A1, *FAM91A1*, p = 8×10^−5^).

### Cisplatin-associated SNPs are enriched for carboplatin-associated SNPs

**Figure 4:**
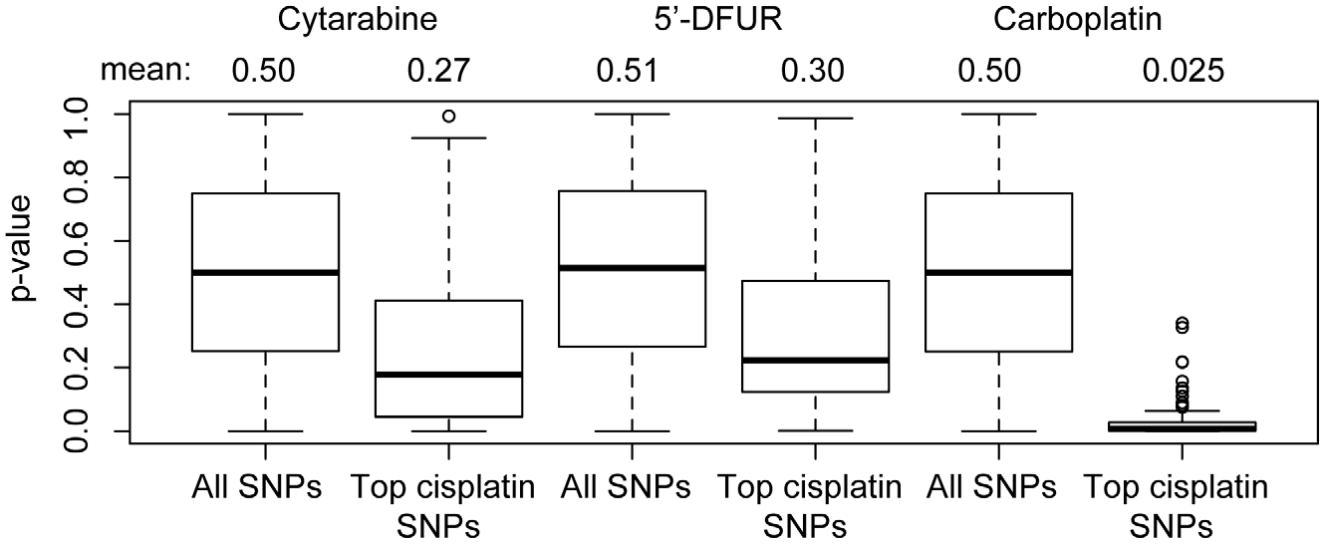
Overall ASW GWAS p-value distributions compared to the p-value distributions of the top cisplatin-associated SNPs. The p-values for the top 190 cisplatin IC_50_-associated SNPs (p≤10^−4^) were pulled from the overall list of ∼2 million SNPs tested for association with each drug-induced phenotype (cytarabine AUC, 5′-DFUR AUC, carboplatin IC_50_). The mean p-value in each class is listed across the top. The boxes define the interquartile range and the thick line is the median. Open dots are possible outliers. The means of the “All SNPs” classes differed from the means of the “Top cisplatin SNPs” classes for all three drugs (cytarabine p = 4.5×10^−24^, 5′-DFUR p = 4.4×10^−24^, carboplatin p = 2.2×10^−191^, Student's t-test).

To compare the top SNP associations for different drug phenotypes, we examined the p-value distributions of the top 190 cisplatin IC_50_ SNPs (p≤10^−4^) in the other three sets of GWA results. That is, we pulled the p-values for these 190 SNPs from the 5′-DFUR AUC, cytarabine AUC and carboplatin IC_50_ datasets and compared the p-value distribution of this subset to the entire p-value distribution (>2 million SNPs) for each drug (Figure 4). The most dramatic shift was seen for carboplatin IC_50_: the overall p-value mean was 0.50 whereas the cisplatin-associated SNP subset p-value mean was 0.025 (p = 2.2×10^−191^). Therefore, cisplatin-associated SNPs are enriched for carboplatin-associated SNPs and common mechanisms may influence the effect of both related drugs. Less dramatic, but still significant, shifts were seen for cytarabine (overall mean 0.50, cisplatin-associated SNP mean 0.27, p = 4.5×10^−24^) and 5′-DFUR (overall mean 0.51, cisplatin-associated SNP mean 0.30, p = 4.4 × 10^−24^). Similar shifts were observed when different thresholds (from 10^−3^ to 10^−5^) were used to choose the top cisplatin-associated SNPs or when the top carboplatin-associated SNPs were examined in the other three sets of GWA results (data not shown). Cytarabine-associated SNPs were not enriched for 5′-DFUR-associated SNPs (p = 0.99). The correlations (Pearson's R) of the four drug phenotypes are shown in Table 1.

**Table 1:** Chemotherapuetic-induced cytotoxicity phenotypic Pearson correlations in the ASW.

	cytarabine AUC	5′-DFUR AUC	carboplatin IC_50_	cisplatin IC_50_
cytarabine AUC	1	−0.11	0.23	0.29
5′-DFUR AUC	−0.11	1	0.26	0.38
carboplatin IC_50_	0.23	0.26	1	0.67
cisplatin IC_50_	0.29	0.38	0.67	1

### Gene associations with chemotherapeutic-induced cytotoxicity

Traditional GWA studies are designed to find individual SNPs with relatively large effects, especially in cohorts of relatively small sample size such as the ASW. They are not designed to detect multiple smaller effects working in concert. If a gene contains more than one variant affecting phenotype, multiple SNPs within the gene or gene region may show marginal levels of significance. The versatile gene-based association study (VEGAS) software was developed to detect these effects by combining the contribution of all SNPs in a gene into a test-statistic and correcting for linkage disequilibrium by using simulations from the multivariate normal distribution [35]. In our implementation of VEGAS, the SNP set for each gene included any SNPs within the gene as well as SNPs 50 kb upstream of the start position and 50 kb downstream of the stop position. The top 10% most significant SNPs for each gene from the initial GWA studies were used in the test-statistic calculation.

**Figure 5:**
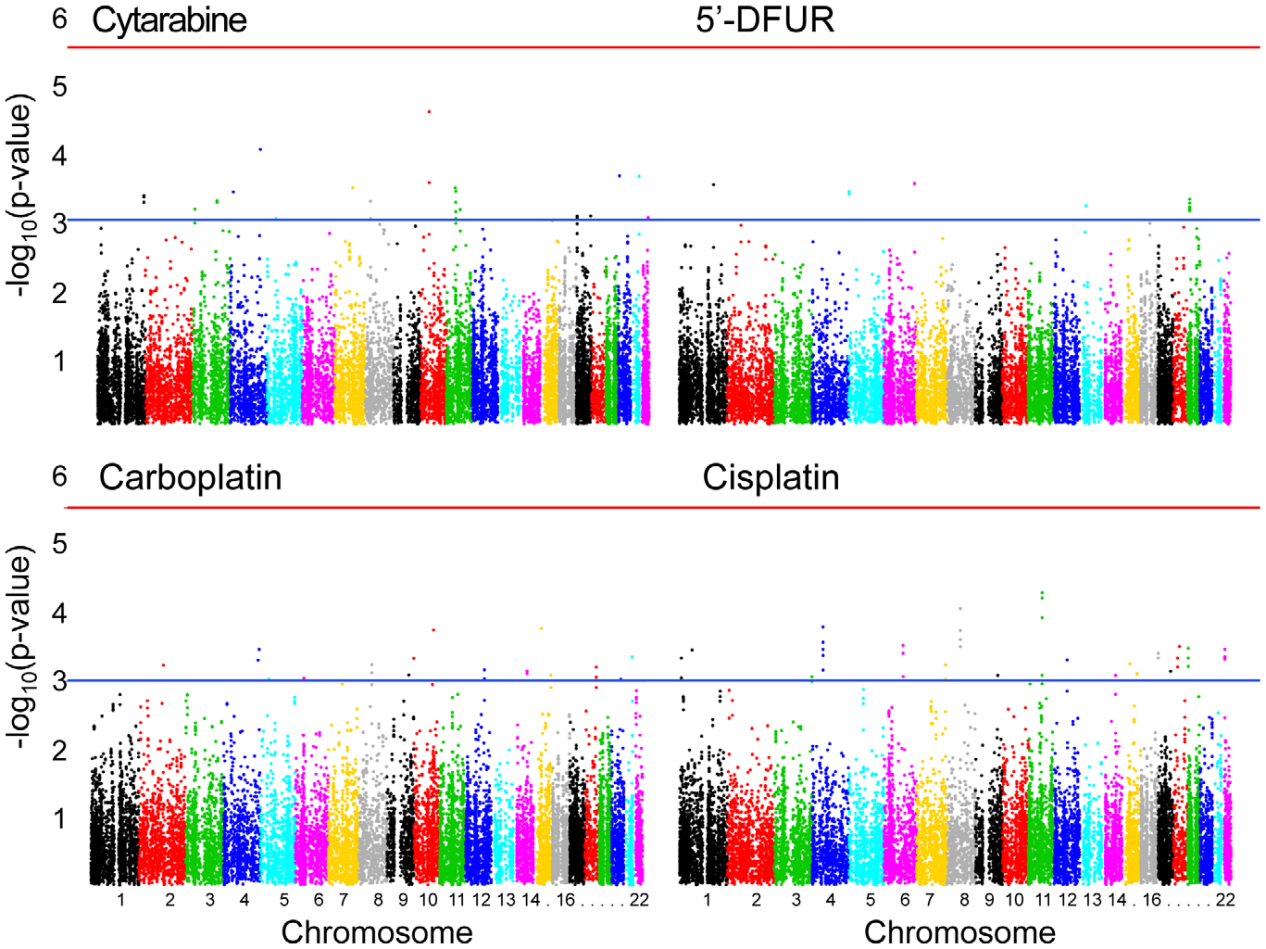
Gene-based genome-wide association results for chemotherapeutic-induced cytotoxicity in the ASW. Each point represents a gene. Blue lines are at the suggestive significance threshold of p = 10^−3^. Red lines are at the Bonferroni-adjusted genome-wide significance threshold of p = 2.8×10^−6^ for 17,723 tests.

**Figure 6:**
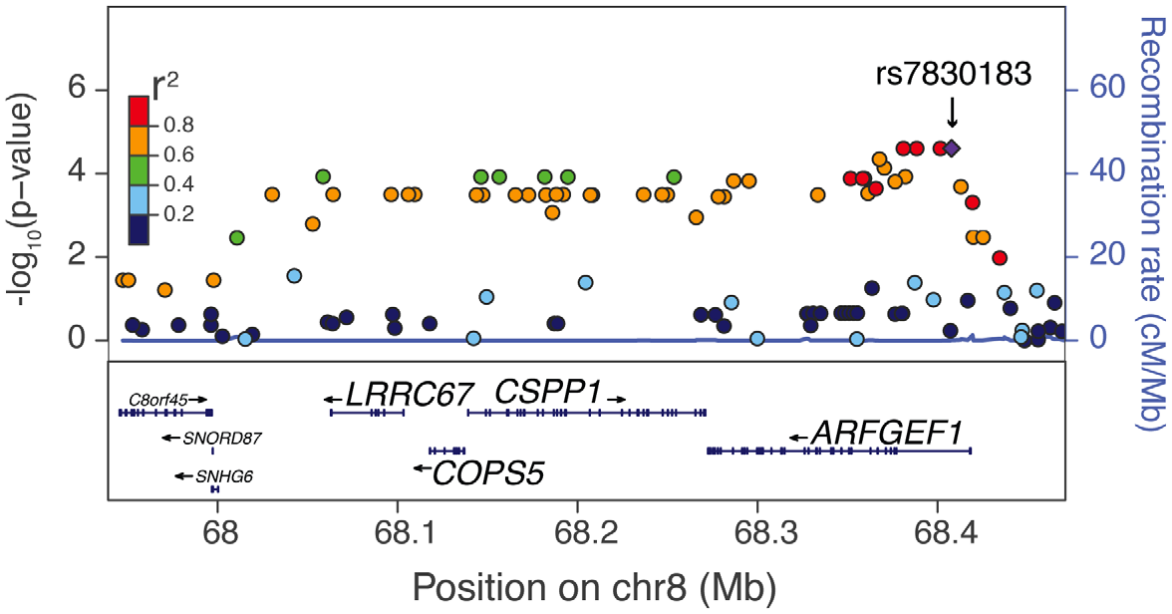
Chromosome 8 cluster of linked genes associated with cisplatin IC_50_ in ASW and YRI. The four genes associated with cisplatin IC_50_ were detected using a gene-based analysis [35]. They are *COPS5* (ASW p = 1.8×10^−4^, YRI p = 0.020), *ARFGEF1* (ASW p = 8.6×10^−5^, YRI p = 0.017), *CSPP1* (ASW p = 2.4×10^−4^, YRI p = 0.017), and *LRRC67* (ASW p = 3.1×10^−4^, YRI p = 0.020). Plots were made with LocusZoom [67] and the position of each dot corresponds to the SNP's p-value for association with carboplatin IC_50_ in the ASW and the color of each dot represents the SNP's linkage disequilibrium r^2^ in the YRI with the labeled SNP (purple diamond).

In total, 17,723 genes annotated in the VEGAS software were tested for association with each of the four drug-induced phenotypes. At the suggestive threshold of p≤10^−3^, 26 genes associated with cytarabine AUC, 11 genes with 5′-DFUR AUC, 20 genes with carboplatin IC_50_, and 41 genes with cisplatin IC_50_ (Figure 5, Table S2). We attempted to replicate these findings by testing each of these 98 genes for association with its respective phenotype in the YRI using the same VEGAS method. Fourteen genes associated with the same drug cytotoxicity phenotype in the YRI (p<0.05) as in the ASW discovery (Table 2). Genes that associated with cytarabine AUC included tumor protein p53 inducible protein 11 (*TP53I11*, ASW p = 3.3×10^−4^, YRI p = 1.3×10^−3^), calcitonin receptor (*CALCR*, ASW p = 3.3×10^−4^, YRI p = 0.038), stathmin-like 4 (*STMN4*, ASW p = 5.2×10^−4^, YRI p = 0.046), and kynurenine 3-monooxygenase (*KMO*, ASW p = 5.4×10^−4^, YRI p = 0.018). Three genes associated with carboplatin IC_50_ in both populations: SNF1-like kinase (*SNF1LK*, ASW p = 4.4×10^−4^, YRI p = 0.028), ADP-ribosylation factor guanine nucleotide-exchange factor 1 (*ARFGEF1*, ASW p = 5.7×10^−4^, YRI p = 0.030), and COP9 constitutive photomorphogenic homolog subunit 5 (*COPS5*, ASW p = 7.5×10^−4^, YRI p = 0.023). *ARFGEF1* (ASW p = 8.6×10^−5^, YRI p = 0.017) and *COPS5* (ASW p = 1.8×10^−4^, YRI p = 0.020) also associated with cisplatin IC_50_, as did centrosome and spindle pole associated protein 1 (*CSPP1*, ASW p = 2.4×10^−4^, YRI p = 0.017) and leucine rich repeat containing 67 (*LRRC67*, ASW p = 3.1×10^−4^, YRI p = 0.020). These four genes cluster together on chromosome 8 and the SNPs within them are in linkage disequilibrium (Figure 6). Three additional genes associated with cisplatin IC_50_ in both populations: PR domain containing 7 (*PRDM7*, ASW p = 3.9×10^−4^, YRI p = 0.033), growth arrest specific 8 (*GAS8,* ASW p = 4.6×10^−4^, YRI p = 0.041), and organic solute transporter alpha (*OSTalpha,* ASW p = 8.7×10^−4^, YRI p = 0.014).

**Table 2:** Genes associated with chemotherapeutic-induced cytotoxicity in the ASW gene-based association studies (p≤10^−3^) that replicated in the YRI (p<0.05).

Phenotype	Chr	Gene	nSNPs	Start Position	Stop Position	ASW Test Statistic	ASW p-value	YRI Test Statistic	YRI p-value
cytarabine AUC	11	*TP53I11*	122	44910474	44929184	309.0	3.3×10^−4^	307.0	1.3×10^−3^
cytarabine AUC	7	*CALCR*	264	92891734	93041686	706.7	3.3×10^−4^	549.9	3.8×10^−2^
cytarabine AUC	8	*STMN4*	89	27149730	27171820	272.7	5.2×10^−4^	100.2	4.6×10^−2^
cytarabine AUC	1	*KMO*	121	239762302	239825567	228.6	5.4×10^−4^	280.5	1.8×10^−2^
carboplatin IC_50_	21	*SNF1LK*	108	43658826	43671430	284.4	4.4×10^−4^	388.0	2.8×10^−2^
carboplatin IC_50_	8	*ARFGEF1*	63	68272450	68418466	311.9	5.7×10^−4^	200.7	3.0×10^−2^
carboplatin IC_50_	8	*COPS5*	18	68117868	68137116	83.5	7.5×10^−4^	81.8	2.3×10^−2^
cisplatin IC_50_	8	*ARFGEF1*	63	68272450	68418466	435.4	8.6×10^−5^	192.1	1.7×10^−2^
cisplatin IC_50_	8	*COPS5*	18	68117868	68137116	165.7	1.8×10^−4^	63.3	2.0×10^−2^
cisplatin IC_50_	8	*CSPP1*	39	68139156	68271050	361.0	2.4×10^−4^	164.0	1.7×10^−2^
cisplatin IC_50_	8	*LRRC67*	21	68062920	68103340	143.5	3.1×10^−4^	67.8	2.0×10^−2^
cisplatin IC_50_	16	*PRDM7*	47	88650474	88669839	117.2	3.9×10^−4^	105.0	3.3×10^−2^
cisplatin IC_50_	16	*GAS8*	58	88616508	88638880	127.8	4.6×10^−4^	118.0	4.1×10^−2^
cisplatin IC_50_	3	*OSTalpha*	53	197427779	197444698	120.1	8.7×10^−4^	141.5	1.4×10^−2^

## Discussion

African American populations are underrepresented in genetic studies, especially those performed on a genome-wide scale. Several studies have indicated an ancestry difference in outcome among patients with various cancer types [7], [20], [23], [24], [33]. We investigated the genetics of chemotherapeutic susceptibility in 83 LCLs derived from the HapMap ASW population. Using local ancestry covariates [38] to account for population structure in the ASW, we tested over 2 million SNPs and identified 325 that were associated with cytarabine AUC, 176 with 5′-DFUR AUC, 240 with carboplatin IC_50_, and 190 with cisplatin IC_50_ (p≤10^−4^). Using a gene-based GWA approach, we identified several suggestive candidate genes: 26 genes for cytarabine, 11 genes for 5′-DFUR, 20 genes for carboplatin and 41 genes for cisplatin susceptibility (p≤10^−3^). Fourteen of these genes showed evidence of replication in the YRI (p<0.05).

One important reason for studying populations of African descent is that common alleles present in African populations may be rare or absent in other populations. For example, the minor allele of the highlighted SNP rs9828664, which has a frequency of 0.29 and associated with increased carboplatin sensitivity in ASW (Figure 3C), is not present in the HapMap population of European ancestry from Utah (CEU). Of the 919 unique SNPs suggestively associated with one or more chemotherapeutic-induced phenotypes in ASW (p≤10^−4^), 237 (25.8%) of them have a minor allele frequency less than 0.05 in the CEU. The minor allele frequency in the CEU is zero for 116 (12.6%) of the ASW-associated SNPs. All of these potential associations would have been missed if only European populations were studied.

As might be expected based on the observation that carboplatin and cisplatin phenotypes were correlated, we observed an enrichment of carboplatin-associated SNPs in the results of cisplatin-associated SNPs. Both carboplatin and cisplatin are platinating agents that act through the formation of intrastrand and interstrand cross-links on DNA, which result in DNA strand breaks leading to cell death [30]. The two agents are often used interchangeably, especially in the treatment of ovarian and lung cancers [26]. These results support that common genetic mechanisms may influence the effects of both drugs. In contrast, a smaller shift in mean p-value was found when comparing the overall p-value distribution to the cisplatin-SNP p-value distribution for unrelated drugs cytarabine and 5′-DFUR even though both phenotypes are also correlated with cisplatin, albeit to a lesser degree (Table 1). Studies in larger cohorts are needed to define common genetic mechanisms within and among chemotherapeutic classes.

The VEGAS gene-based method revealed two linked genes associated with both carboplatin- and cisplatin-induced cytotoxicity in ASW and YRI. One of these genes, *COPS5*, is also know as *JAB1* (Jun activation domain-binding protein 1) and encodes a protein involved in multiple signaling pathways [41]. Overexpression of *COPS5* has been implicated in the pathogenesis of several types of cancer in humans and in some cases has correlated with poor prognosis [42]–[46]. In one study, loss of *COPS5* expression sensitized both mouse primary embryonic fibroblasts and osteosarcoma cells to radiation-induced apoptosis [41]. *COPS5* is linked to three other genes on chromosome 8 that also associated with cisplatin IC_50_ in the gene-based analysis (Figure 6). The p-values of two of these three genes for association with carboplatin IC_50_ were just above our suggestive threshold of p≤10^−3^. Although *COPS5* is the only gene of these four known to be involved in tumorigenesis, the possibility that the others are involved in susceptibility to platinating agents cannot be ruled out due to the strong linkage disequilibrium in the region.

Two of the genes that associated with chemotherapeutic-induced cytotocity in the gene-based analysis in both populations are candidate tumor suppressors. *TP53I11* associated with cytarabine AUC and overexpression of the gene promotes apoptosis in hepatocellular carcinoma cells [47]. *GAS8*, which associated with cisplatin IC_50_, is sometimes deleted in breast and prostate cancer [48], [49]. Further studies are needed to elucidate how the function of all fourteen of the genes identified in our gene-based genome-wide analysis may affect tumor cell response to their associated chemotherapeutic agents.

Our approach to correct for population structure in the admixed ASW population differs from that taken in other African American GWA studies [4], [50]. Rather than using principal components to infer global ancestry, we used HAPMIX to infer local ancestry at each SNP locus and included this value as a covariate in the association test [38]. Similar local ancestry approaches were taken in candidate gene studies using Ancestry Informative Markers [51], [52]. Using local ancestry rather than global ancestry for African Americans more accurately reflects the structure of the population at each SNP locus and is thus useful for the single-marker tests of GWA studies. Recently, it was shown that combining local ancestry and admixture association signals into a test statistic had more power to map disease loci than case-control studies correcting for global ancestry only in African American populations [53]. HAPMIX is one of several methods for detecting chromosomal segments of distinct ancestry [54]–[56] and determining the best way to apply local ancestry in genetic association tests in admixed populations remains an open question.

Currently, GWA studies in populations of African descent are limited by SNP genotyping arrays, which were designed to capture genetic variation in European populations where LD blocks are larger [3]. Thus, SNP tagging does not work as well in populations of African ancestry compared to non-African populations. It is also more difficult to impute genotypes for African American samples than more genetically homogeneous populations, although using a pooled reference panel as was done here with YRI and CEU has been shown to boost performance [57]. Furthermore, because the LD patterns among variants are less consistent between African subgroups, it can be more difficult to replicate associations once they have been detected, unless the causal variant has been identified [58]. Therefore, we took a gene-based approach to attempt to replicate our initial ASW findings in the YRI. We found evidence of replication for 14 of the 98 genes identified in the ASW using the gene-based GWA approach, but larger sample sizes are need to confirm these associations.

Whole genome sequencing will shift association studies from LD-tagged variation to directly genotyped variation, which will benefit populations with African ancestry. The ASW population is in the pipeline to be sequenced by the 1000 Genomes Project [59], [60]. In our gene-based analysis, we only considered SNPs within a gene region, but *trans* eQTLs are also known to affect gene activity [40], [61], [62]. In future analyses, we plan to extend the VEGAS method to incorporate additional SNPs discovered by the 1000 Genomes Project and SNPs associated with gene expression in relevant tissues into the gene-based test. Our results highlight the importance of studying populations of African descent and we eventually hope to clinically validate both genes and variants in a cohort of African American patients treated with one or more of the chemotherapeutic agents studied to determine their roles in patient response and toxicity.

## Materials and Methods

### Lymphoblastoid Cell Lines

International HapMap Project EBV-transformed LCLs from 83 individuals of African ancestry from the Southwestern United States (ASW [HAPMAPPT07]) and 176 individuals from the Yoruba in Ibadan, Nigeria (YRI [HAPMAPPT03 and HAPMAPPT04] were purchased from the Coriell Institute for Medical Research. The family structure of the ASW population was 10 trios (mother, father, and child), 20 duos (one parent and child) and 13 unrelated singletons. The family structure of the YRI was 57 trios, 1 duo and 3 singletons. Cell lines were maintained in RPMI 1640 (Mediatech, Herndon, VA, USA) supplemented with 15% fetal bovine serum (HyClone Laboratories, Logan, UT, USA) and 1% L-glutamine (Invitrogen, Carlsbad, CA, USA). Cell lines were passaged 3 times per week at a concentration of 3.5×10^5^ cells/mL and incubated at 37°C with 5% CO_2_ and 95% humidity.

### Chemotherapeutic Drugs and Cytotoxicity Assays

Capecitabine is not activated to 5-fluorouracil in LCLs, therefore 5′-deoxyfluoruridine (5′-DFUR) (an active form of capecitabine) was obtained from LKT Laboratories (St. Paul, MN, USA) and prepared in equal amounts of PBS (Invitrogen, Carlsbad, CA, USA) and DMSO (Sigma, St. Louis, MO, USA) as a stock solution at a concentration of 80 mM. Carboplatin [7], cisplatin [8] and cytarabine [6] were prepared and cells were treated as previously described. Briefly, the cytotoxic effect of carboplatin, cisplatin, cytarabine, and 5′-DFUR was determined using a short-term alamarBlue colorimetric assay (Biosource International, Camarillo, CA). LCLs (>85% viability) were plated in triplicate at a density of 1×10^5^ cells/mL and drug added 24 h after plating. Cells were exposed to cisplatin for 48 h and the other three drugs for 72 h at the following concentrations: 1, 2.5, 5, 10, and 20 µM cisplatin; 5, 10, 20, 40, and 80 µM carboplatin; 1, 5, 10, and 40 µM cytarabine; and 2.5, 10, 20, and 40 µM 5′-DFUR. Final percent survival was ascertained by averaging at least six replicates from two independent experiments. Growth rates for each cell line were estimated by the alamarBlue method, as previously described [12], [63]. The concentration required to inhibit 50% of cell growth (IC_50_) was calculated for each carboplatin- and cisplatin-treated cell line. The area under the survival curve (AUC) was calculated for cytarabine- and 5′-DFUR-treated cell lines using the trapezoidal rule. All IC_50_ and AUC values were adjusted for baseline growth rate and the residuals were rank-transformed to normality before statistical modeling using the *rntransform* function in the *GenABEL* R library.

### Global Ancestry Analysis

We used a linkage disequilibrium-pruned (r^2^>0.3) set of 102,386 SNPs to compare the genotypes of unrelated ASW, YRI (Yoruba from Ibadan, Nigeria), CEU (European descent from Utah) and CHB (Han Chinese from Beijing) individuals. Genotypes came from HapMap release 22 for YRI, CEU and CHB and release 27 for ASW. Principal components were computed using the EIGENSTRAT method to model the ancestry of the individuals in these four populations [37].

### Local Ancestry Analysis

Local ancestry for each ASW individual at each genotyped SNP locus was estimated using the HAPMIX software [38]. Phased genotypes from the HapMap YRI and CEU (release 22) populations were used as the two parental populations to estimate the ancestry of the ASW population (release 27). A total of 1,152,289 SNPs that have a minor allele frequency of at least 5% and are in Hardy-Weinberg equilibrium (p>0.001) in the ASW were used for further analysis. Suggested input parameters for African American populations of 20% European ancestry and 6 generations since admixture were used [38]. For each individual, the algorithm estimated the number of CEU chromosomes (continuous value between 0 and 2) at each SNP locus.

### Genotype Imputation

To increase genome coverage of the ASW, ungenotyped makers were imputed using the BEAGLE software [64]. Both YRI and CEU (HapMap r22) were used as reference populations as in Hao et al. [57]. Beagle imputes ungenotyped markers for unrelateds, parent-offspring pairs and parent-offspring trios by modeling the family structure in the analysis. We also used BEAGLE to impute markers that were not genotyped in YRI phase 3 (r27) by using YRI phase 2 (r22) as reference. To measure the accuracy of the imputation at each SNP locus, R^2^ was calculated as described following 100 imputations of the data [64]. Of 1,303,563 SNPs for which imputation was attempted in the ASW, 1,064,943 (81.7%) had an imputation R^2^>0.80. Imputed SNP genotypes with R^2^>0.80, minor allele frequency>0.05, no Mendelian errors and in Hardy-Weinberg equilibrium (p>0.001) were carried through the rest of the analysis. Imputation increased the number of SNPs tested in the ASW GWAS from 1,152,289 to 2,217,232. Local ancestry for each imputed SNP was inferred by using the predicted number of CEU chromosomes from nearest genotyped SNP.

### GWA Analysis

We performed GWA studies in the ASW population between each rank-transformed drug phenotype adjusted for growth rate and greater than 2 million SNPs using the quantitative trait disequilibrium test (QTDT) total association model [65]. Local ancestry (predicted number of CEU chromosomes at each locus) was included as a covariate in the model for each drug. Two of the phenotypes had genomic control lambda (λ_GC_) values [66] greater than 1 (1.11 for cytarabine AUC and 1.23 for carboplatin IC_50_). The λ_GC_ is computed as the median of all genome-wide observed test statistics (chi-square statistics) divided by the expected median of the test statistic under the null hypothesis of no association (making the assumption that the number of true associations is very small compared to the millions of tests performed). The cytarabine and carboplatin results were corrected for residual inflation of the test statistic by dividing the observed test statistic at each SNP by the λ_GC_ [66] and then carried through subsequent analyses. The 5′-DFUR and cisplatin GWA results adjusting for local ancestry only were not inflated (λ_GC_ = 0.94 and 0.96, respectively) and no correction was used. Q-Q plots of the corrected models are shown in Figure S1.

We considered chemotherapeutic susceptibility-associated SNPs with p≤10^−4^ as strongly suggestive and SNPs with p≤5×10^−8^ as genome-wide significant. We chose p≤10^−4^ as suggestive because previous work has shown that SNPs associated with chemotherapeutic drug susceptibility at this threshold and below are enriched for eQTLs, an important functional class [39]. The complete GWA analysis for 176 YRI cell lines has been performed between each rank-transformed drug phenotype adjusted for growth rate and greater than 2 million SNPs using the QTDT [65] total association model (Amy L. Stark, Heather E. Wheeler, R. Stephanie Huang, M. Eileen Dolan, unpublished data). We used the YRI GWA results in the gene-based GWA analysis when testing the 98 genes found in the ASW for replication in the YRI.

### Gene-based GWA Analysis

The versatile gene-based association study (VEGAS) method is a gene-level analysis that can detect genes associated with a trait due to multiple SNPs of relatively small effect [35]. We tested the 17,723 genes annotated by the VEGAS software for association with each of the four drug phenotypes in the ASW population using the results from the initial SNP-based GWAS. The VEGAS software combines the contribution of all SNPs in a gene into a test-statistic and corrects for linkage disequilibrium in each population by using simulations from the multivariate normal distribution [35]. In our implementation of VEGAS, the SNP set for each gene included any SNPs within the gene as well as SNPs within 50 kb upstream or downstream of the gene. The top 10% most significant SNPs from the SNP set of each gene were used in the test-statistic calculation. Similar results were observed when all the SNPs in the SNP set of each gene were used in the analysis (Table S2). Up to 10^6^ multivariate normal vectors of the appropriate SNP set size for each gene were simulated, and the empirical gene-based p-value was calculated as the proportion of simulated test statistics that exceeded the observed gene-based test statistic. After completing the ASW VEGAS analysis, we tested the top 98 genes (p≤10^−3^) for association with the respective phenotype in the YRI population.

## Supporting Information

Figure S1 **Q-Q plots of GWA results for chemotherapeutic-induced cytotoxicity in the ASW.** Cytarabine and carboplatin results were adjusted using the genomic control method. 5′-DFUR and cisplatin results were not adjusted.
(TIF)

Table S1 **Top ASW drug-induced cytotoxicity-associated SNPs (p≤10^−4^).**
(XLS)

Table S2 **Top ASW drug-induced cytotoxicity-associated genes (p≤10^−3^).**
(XLS)
