# Supplementary figures and images for: Genome-Wide Local Ancestry Approach Identifies Genes and Variants Associated with Chemotherapeutic Susceptibility in African Americans
Source: PLoS One. 2011 Jul 6;6(7):e21920. doi: 10.1371/journal.pone.0021920 (PMC3130766; doi:10.1371/journal.pone.0021920)

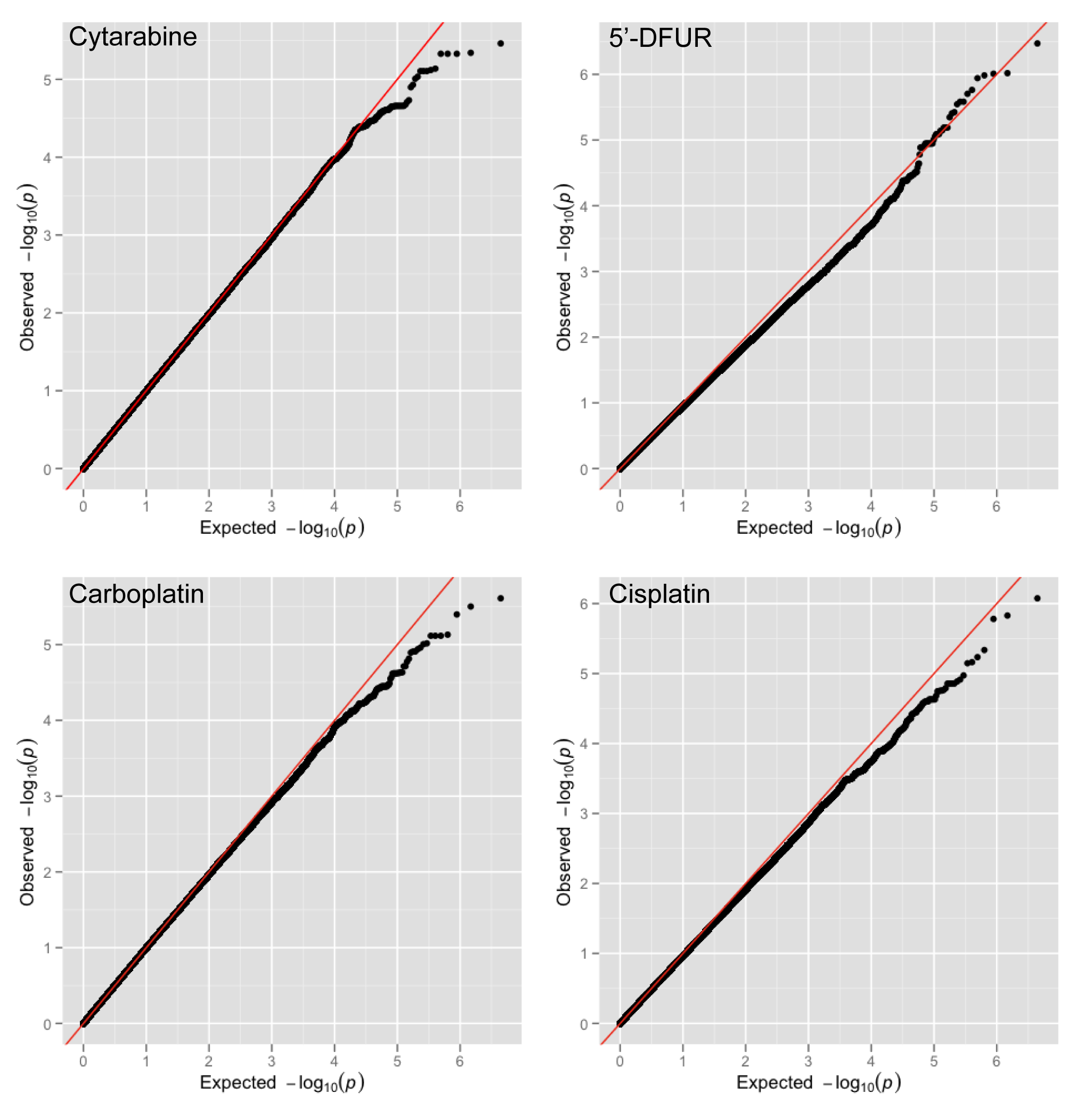

Supplement: Figure S1 — Q-Q plots of GWA results for chemotherapeutic-induced cytotoxicity in the ASW. Cytarabine and carboplatin results were adjusted using the genomic control method. 5′-DFUR and cisplatin results were not adjusted. (TIF) [file pone.0021920.s001.tif]
